# The usefulness of flexible cystoscopy for preventing double-J stent malposition after laparoscopic ureterolithotomy

**DOI:** 10.1186/s12894-017-0232-4

**Published:** 2017-06-15

**Authors:** Jae-Yoon Kim, Seok-Ho Kang, Jun Cheon, Jeong-Gu Lee, Je-Jong Kim, Sung-Gu Kang

**Affiliations:** Department of Urology, Korea University College of Medicine, 73 Inchon-Ro, Sungbuk-gu, Seoul, 136-705 Republic of Korea

**Keywords:** Laparoscopy, Stone disease, Ureteral calculus, Ureteral stent

## Abstract

**Background:**

The aim of this study was to evaluate the role of flexible cystoscopy in preventing malpositioning of the ureteral stent after laparoscopic ureterolithotomy in male patients.

**Methods:**

From April 2009 to June 2015, 97 male patients with stones >1.8 cm in the upper ureter underwent intracorporeal double-J stenting of the ureter after laparoscopic ureterolithotomy performed by four different surgeons. In the last 50 patients who underwent laparoscopic ureterolithotomy flexible cystoscopy was performed through the urethral route to confirm the position of the double-J stent, while in the first 47 correct positioning of the stent was confirmed through postoperative KUB. The demographic data and perioperative outcomes were reviewed retrospectively. Penalized logistic regression analysis was used to evaluate the effects of flexible cystoscopy.

**Results:**

Upward malpositioning of the ureteral stent was found in 9 of the 47 (19.1%) patients who underwent surgery without flexible cystoscopy. Among the 50 most recent patients who underwent surgery with flexible cystoscopy through the urethral route, upward malpositioning was observed in 10 (20%) patients. The factors preventing upward malpositioning of the double-J catheter in multivariate analysis were surgeon (*p* = 0.039) and use of flexible cystoscopy (*p* = 0.008).

**Conclusion:**

Flexible cystoscopy is a simple, safe, quick, and effective method to identify and correct malpositioning of double-J stents, especially in male patients.

**Trial registration:**

This study was registered with ClinicalTrials.gov Registry on May 11, 2017 (retrospective registration) with a trial registration number of NCT03150446.

**Electronic supplementary material:**

The online version of this article (doi:10.1186/s12894-017-0232-4) contains supplementary material, which is available to authorized users.

## Background

The treatment of large upper ureteral stones is still controversial [1, 2]. The American Urological Association (AUA) and the European Association of Urology (EAU) recommend that laparoscopic stone removal may be considered in rare cases in which shockwave lithotripsy (SWL), ureteroscopic lithotripsy (URS), and percutaneous nephrolithotomy fail or are unlikely to be successful [1–5]. In a recent meta-analysis of treatment of large proximal ureteral stones, Torricelli et al. reported that the outcomes of laparoscopic ureterolithotomy (LUL) for larger upper ureteral stones are favorable compared with those of URS, and LUL should be considered as a first-line option when flexible ureteroscopy is not available [6]. After such surgery, many surgeons prefer placing a double-J stent, a ureteral catheter that is passed through the ureter from the kidney to the bladder [7, 8]. Although double-J stent placement after LUL remains controversial, many urologists believe that it may help prevent postoperative urinary leakage [9].

Intracorporeal double-J stenting is technically difficult, and malpositioning often occurs after surgery in clinical practice [10]. However, the actual rate of malpositioning of stents has not been reported yet. Although clinicians use different ways to place double-J stents precisely, accurate stent placement before the closure of the ureteral incision might be difficult to confirm.

Upward malpositioning of the stent after surgery may necessitate removal of the stent using a ureteroscope. It is difficult to remove stents in the outpatient setting without anesthesia to reduce pain and discomfort, especially in male patients.

In this study, we used flexible cystoscopy through the urethral route before closure of the ureteral incision to confirm that the double-J stent was placed correctly in the bladder of male patients. Upon identification of upward malpositioning of the ureteral stent, position adjustments were performed by intracorporeally manipulating the ureteral stent through the incision site of the ureter. The aim of this study was to determine the malpositioning rate and predicting factors associated with upward malpositioning of intracorporeal double-J stents after LUL and to evaluate the usefulness of flexible cystoscopy in preventing such malpositioning in male patients.

## Methods

**Fig. 1:**
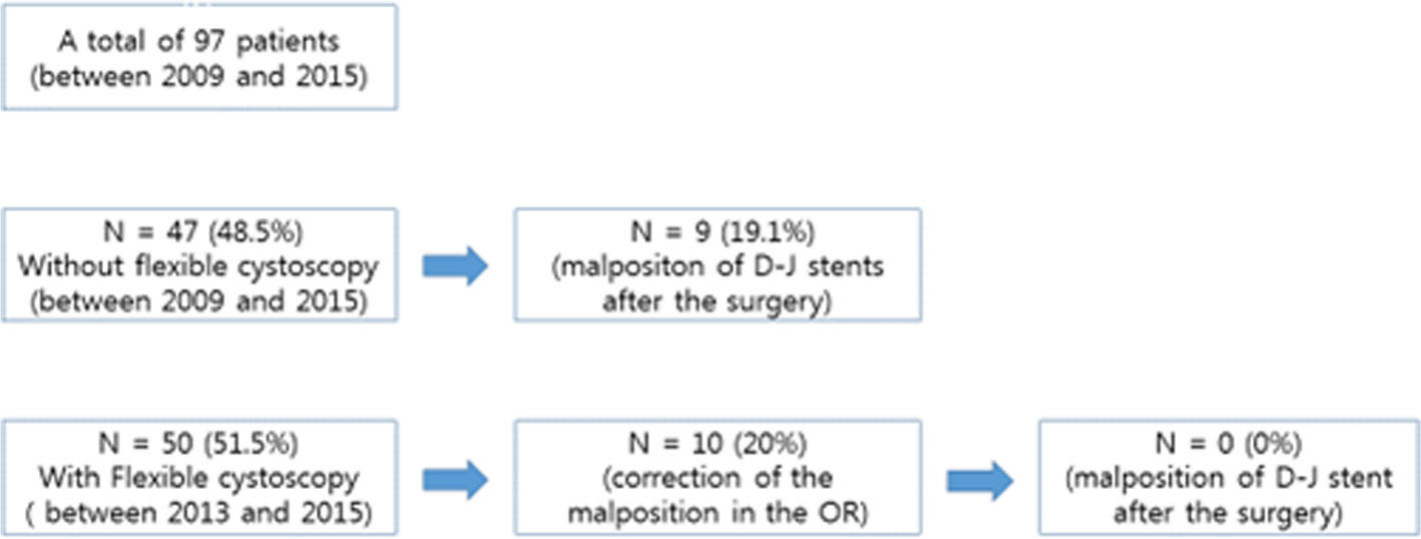
The flowchart of the study

From April 2009 to June 2015, a total of 97 male patients with large stones (>1.8 cm in size) of the upper ureter underwent LUL. In all patients, intracorporeal double-J stents were placed after surgery. In the first 47 patients, the surgery was finished without verification of double-J stent placement (this was done on postoperative imaging). In the latest 50 consecutive patients, flexible cystoscopy was performed through the urethral route before closure of the ureteral incision to determine whether the double-J stent was correctly placed in the bladder (Fig. 1).

Patient demographic data such as age, height, weight, body mass index, stone level, stone size, degree of hydronephrosis, and previous ureteric procedures were reviewed retrospectively. The levels and sizes of stones were determined using kidney-ureter-bladder (KUB) radiography or computed tomography. The degree of hydronephrosis was determined using a scale from 0 to 4 according to the Society of Fetal Ultrasound grade system [11]. Perioperative data, including surgeon, surgical approach, and use of flexible cystoscopy, were also collected retrospectively. Perioperative outcomes such as operative time, upward malpositioning rate, and additional time for flexible cystoscopy were reviewed. We defined upward malpositioning as placement of the double-J stent such that its tip is straight instead of being curled on postoperative KUB radiography. Accordingly, we reviewed all postoperative follow-up KUB images.

To identify factors predicting malpositioning, logistic regression analysis was conducted with SPSS, version 22.0. To evaluate the effects of flexible cystoscopy in reducing the malpositioning rate, penalized logistic regression analyses were performed with SAS 9.4, with a *p* < 0.05 considered to represent a statistically significant difference.

### Intracorporeal double-J stent insertion after laparoscopic ureterolithotomy

After placing the patient in a semilateral position, a skin and fascial incision was made laterally to the rectus muscle at the level of the umbilicus, and a 10-mm balloon trocar was inserted into the abdominal cavity. Subsequently, with the pneumoperitoneum maintained at 12 mmHg using CO_2_, two more trocars (10 and 5 mm) were introduced under laparoscopic view parallel to the first trocar. At the beginning of the procedure, the descending colon was reflected from its retroperitoneal attachment and moved medially to identify the ureter. Each stone was identified as a prominent bulge on a suspicious lesion. To prevent upward movement of the stone, careful dissection was performed while avoiding touching the ureter directly. A needle holder with a broken 15th blade tip was used to incise the ureter overlying the stone, which enabled a sharp, precise ureteral incision at the level of the stone. Subsequently, the stone was removed with a grasper. The ureter was then catheterized using a standard 6F double-J stent with both long and short guidewires inserted through two separate side holes of the stent that were closed at both ends. Then, the prepared stent was inserted in a bidirectional manner through the ureterotomy site, and the two guidewires were extracted.

### Laparoscopic adjustment of double-J stent with flexible cystoscopy

**Fig. 2:**
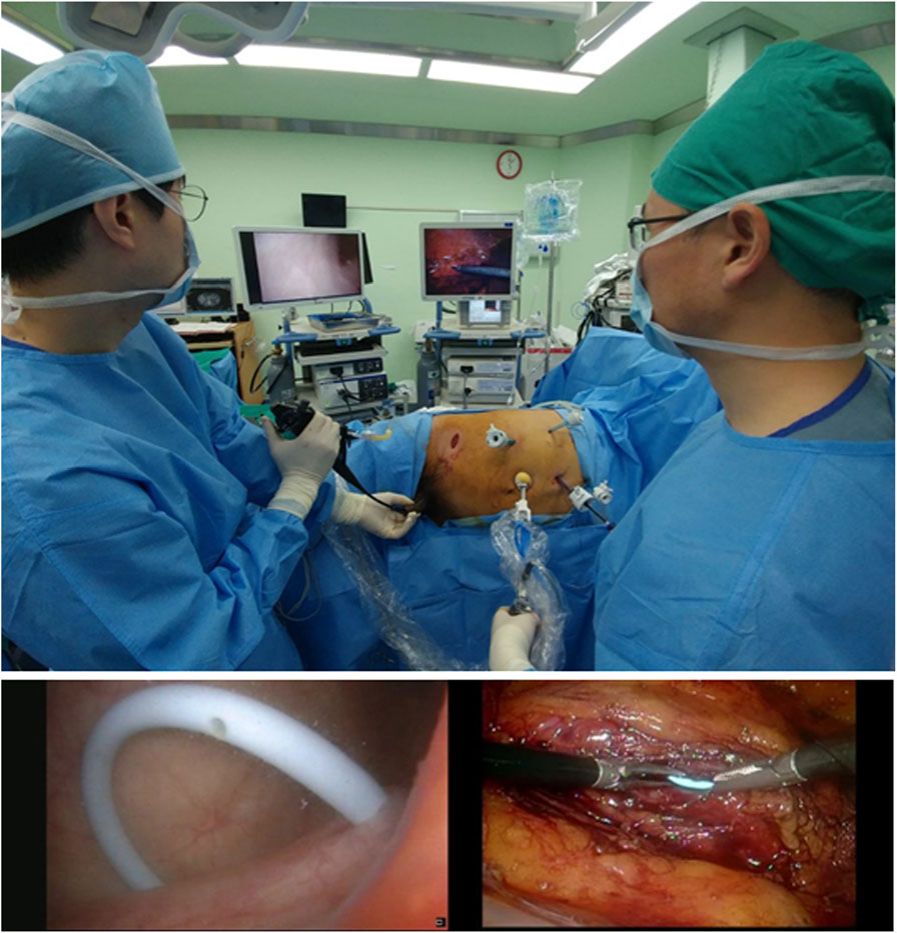
The operating room set-up. The surgeon who manipulates the D-J catheter uses monitor A, while the assistant inserts a flexible cystoscope and verifies if the double-J stent is positioned correctly using monitor B

From April 2013 to June 2015, the last 50 patients with large upper ureteral stones underwent LUL with flexible cystoscopy to confirm the correct positioning of the double-J stent. After intracorporeal insertion of the double-J catheter, additional endoscopic monitoring with flexible cystoscopy was performed. The surgeon manipulating the double-J catheter used monitor A, while an assistant inserted a flexible cystoscope into the bladder through the urethral route and determined whether the double-J stent was correctly placed in the bladder using monitor B before suturing the site of ureterotomy (Fig. 2). If the stent was well-placed, the flexible cystoscope was withdrawn. If the double-J stent was not visualized in the bladder, the surgeon pushed the stent inferiorly using a laparoscopic instrument and monitor A until the stent came out through the ureteral orifice on monitor B (Additional file 1). After placement of the stent, the ureteral incision was closed with 4–0 Vicryl interrupted sutures.

## Results

The preoperative patient demographics are summarized in Table 1. The mean age was 53.46 ± 13.72 years. The mean stone size was 1.87 ± 0.33 cm, and all patients had upper ureteral stones. In 16 (16.5%) cases, the stones were of level L2 or below, whereas in the remaining 81 (83.5%) cases the level exceeded L2. One patient (1%) had hydronephrosis of grade 0, 26 (26.8%) of grade 1, 39 (40.2%) of grade 2, 17 (17.5%) of grade 3, and 14 (14.4%) of grade 4. Medical history review revealed that 23 patients (23.7%) had undergone SWL and 12 patients (12.4%) had undergone URS.

**Table 1:** Patient demographics and clinical characteristics

Variable	Quantity/Value
Age (years)	55 (21–81)
Sex	
Male	97 (100%)
Height (cm)	165.03 ± 10.29 (141–197)
Weight (kg)	68.15 ± 13.17 (38.7–121)
BMI	24.87 ± 3.07 (17.04–32.89)
Stone level	
Upper ureter (groups divided via L2 level)	97 (100%)
L2 level or below	16 (16.5%)
Above L2 level	81 (83.5%)
Stone size (cm)	1.87 ± 0.33 (1.52–2.42)
Degree of hydronephrosis	
Grade 0	1 (1.0%)
Grade 1	26 (26.8%)
Grade 2	39 (40.2%)
Grade 3	17 (17.5%)
Grade 4	14 (14.4%)
Previous history of the ureteral procedure	
None	65 (67.0%)
SWL	23 (23.7%)
URSL	12 (12.4%)

Data are presented as n (%), mean ± SD (range) or median (range)

*BMI* Body Mass Index, *SWL* Shock Wave Lithotripsy, *URSL* Ureteroscopic Lithotripsy

The perioperative data and outcomes are presented in Table 2. The mean operative time was 137.33 ± 52.44 min; 84 patients were treated using the transperitoneal approach and 13 patients using the retroperitoneal approach. The surgeries were performed by four surgeons (40, 26, 20, and 11 cases).

**Table 2:** Perioperative data and outcomes

Variable	Quantity/Value
Operation time (min.)	137 ± 52.44 (45–280)
EBL (ml)	58.6 ± 15.78 (20–90)
Method of surgical approach	
Transperitoneal	84 (86.6%)
Retroperitoneal	13 (13.4%)
Surgeon	
1	40 (41.2%)
2	26 (26.8%)
3	20 (20.6%)
4	11 (11.3%)
Upward malpositioning after surgery without using flexible cystoscopy	9/47 (19.1%)
Flexible cystoscopy use	50/97
Case adjusted by flexible cystoscopy	10/50(20%)
Upward malpositioning after surgery using flexible cystoscope	0/50 (0%)
Mean added time for flexible cystoscopy (min.)	4 min 30s

Data are presented as n (%), mean ± SD (range) or median (range)

*EBL* Estimated Blood Loss

On postoperative KUB radiography, we identified upward malpositioning of ureteral stents in 9 of the 47 (19.1%) patients who underwent surgery without flexible cystoscopy. Among the 50 most recent patients who underwent surgery with flexible cystoscopy through the urethral route, upward malpositioning was identified in 10 (20%). In these 10 patients, the upward malpositioning of the double-J stent was laparoscopically corrected, and there were no patients with upward malpositioning after surgery until the removal of the stents. The mean additional operative time required for flexible cystoscopy was 4 min and 30 s.

In univariate analysis, presence of hydronephrosis (*p* = 0.044) predicted upward malpositioning (Table 3). In multivariate penalized logistic regression analysis, surgeon (0.039) and flexible cystoscopy (0.008) were significant factors preventing malpositioning (Table 4).

**Table 3:** Univariate logistic regression analysis of variables affecting upward malpositioning of ureteral stents (SPSS version 22)

Variable	Odds ratio	95% Confidence Interval	*P*-value
Age	0.979	0.945–1.015	0.246
Height (cm)	0.991	0.945–1.039	0.706
Weight (kg)	1.010	0.975–1.047	0.584
BMI	1.084	0.923–1.274	0.324
Stone level (groups divided via L2 level)	0.379	0.119–1.205	0.100
Stone size (cm)	1.433	0.533–3.849	0.475
Degree of hydronephrosis			0.044
Previous history of the ureteral procedure	0.567	0.187–1.719	0.316
SWL	0.467	0.124–1.755	0.259
URSL	1.241	0.304–5.064	0.764
Operation time (min.)	1.003	0.994–1.012	0.552
Transperitoneal VS retroperitoneal	2.656	0.766–9.207	0.123
Surgeon type			0.077
2	0.338	0.097–1.176	0.088
3	0.098	0.012–0.809	0.031
4	0.413	0.078–2.180	0.297
Flexible cystoscopy use	0	0	0.997

*BMI* Body Mass Index, *SWL* Shock Wave Lithotripsy, *URSL* Ureteroscopic Lithotripsy

**Table 4:** Penalized logistic regression analysis of variables affecting upward malpositioning of ureteral stents (SAS version 9.4)

Variable	Odds ratio	95% Confidence Interval	*P*-value
Surgeon			0.039
Flexible cystoscopy use	0.02	<0.001–0.35	0.008

## Discussion

Several modalities are available for the treatment of large upper ureteral stones [12–17]. Although controversial, SWL and URS have been recommended by the AUA and EAU guidelines as the first choice for proximal large ureteral stones, whereas LUL has been used as one of the options in the management of upper urinary tract stones [5, 6]. With regard to the dimension of large proximal ureteral stones, it seems that there is no clear definition. In a recent meta-analysis, Torricelli et al. analyzed six randomized controlled trials for large upper ureteral stones. These six studies used different inclusion criteria in terms of stone size: two studies used 10 mm [13, 16], whereas the other three used 12 mm [17], 15 mm [12], and 20 mm [14]. In our study, we analyzed patients with stones exceeding 15 mm in size. The main advantage of LUL is the high probability of removing impacted stones in one session without additional procedures, whereas SWL and ureteroscopic approaches are characterized by higher risks of remnant stones, stone-free failure (especially in cases of large stones), impacted stones, and hard stones.

Although many surgeons prefer to insert double-J stents after LUL, there is controversy about whether ureteral stenting is necessary. Hammady et al. [18] performed a randomized controlled study and concluded that LUL without stent insertion is safe, cost-effective, and relatively quick. In addition, LUL without stenting does not require auxiliary procedures for removing the stent afterwards. On the other hand, Karami et al. [9] reported that placing a stent during LUL does not increase the operation time and may play an important role in preventing urinary leakage. In our study, there were no patients with flank pain or increased postoperative drainage after LUL with double-J stent placement.

The procedures used to insert the double-J stent intracorporeally after LUL are challenging and time consuming for inexperienced surgeons [10]. Therefore, various methods have been described to accomplish this successfully. In one of the most commonly used techniques, a retrograde double-J stent is placed beneath the stone using cystoscopy before LUL and advanced after stone removal [19–24].

Chen et al. [10] evaluated the feasibility of ureteroscope-assisted ureteral double-J stenting after LUL and found that it was a simple and safe alternative method of correct stent placement. However, retrograde cystoscopic or ureteroscopic stenting requires additional position changes. Moreover, cystoscopic retrograde stenting is associated with risks due to advancing the suture site without direct visualization. Alongside these retrograde cystoscopic or ureteroscopic stenting techniques, several intracorporeal stenting techniques have previously been described [25–28]. However, these techniques require fluoroscopic confirmation of correct placement after closing the ureter. It is difficult to place the film between the patient’s body and the operation table to take an intraoperative KUB image when the patient is draped. Additionally, it takes substantial time to obtain KUB radiography results. Moreover, upon recognition using KUB radiography and correction of a malpositioned double-J stent, another KUB image would be necessary to confirm correct positioning. Therefore, readjustment of the stent using intraoperative KUB radiography for guidance may be technically difficult and increase operative time, potentially leading to stent failure. In this regard, our technique does not require position changes or fluoroscopic confirmation. Furthermore, the additional time required for flexible cystoscopy was <2 min if the stent was correctly placed in the bladder. Even when the stent was not adequately placed in the bladder, the mean additional time for readjustment was only 4 min. Flexible cystoscopy is an especially suitable method for male patients who might experience moderate pain if ureteroscopic removal of the stent is needed after LUL because of its upward malpositioning.

Surgeon and use of flexible cystoscopy were significant predicting factors for upward malpositioning. To our knowledge, no previous studies related to malpositioning of double-J stents have addressed this question. It makes intuitive sense that the rate of malpositioning differs according to the operator. Experience of the surgeon is important in preventing malpositioning. However, all our surgeons had several cases of upward malpositioning. Additionally, the use of flexible cystoscopy resulted in a 100% success rate, and malpositioning was corrected with a *p*-value of 0.008 in our study.

We recognize several limitations in this study. The first is that the data were collected from four different surgeons with different surgical experience without randomization. The differences in the number of performed LUL among the surgeons may indicate different levels of experience, resulting in variations in operative time, blood loss, and complication rates.

The second limitation is a lack of precise definition of upward malpositioning of the double-J stent. In fact, criteria for deciding whether the double-J stent is accurately positioned have not been defined in any previous study. Additionally, in this retrospective study, methods for the removal of the double-J stent after LUL were not described in the medical records in many cases. Therefore, we defined upward malpositioning as placement of the double-J stent such that its tip is straight instead of being curled on postoperative KUB radiography. This strict definition may have contributed to the high rate of malpositioning of double-J stents in this study. This rate would be lower if malpositioning was defined to occur if the double-J stent had to be removed with an ureteroscope after LUL. In fact, flexible cystoscopy through the urethral route determined that the double-J stent was malpositioned in 10 of 50 cases (20%), which supports our assertion that the malpositioning rate in many cases is actually higher.

The third limitation of our study is the small sample size. Thus, future studies with a larger number of patients are needed.

Despite these limitations, our results show that flexible cystoscopy may be a useful method for confirming the placement of ureteral stents in the bladder and reducing malpositioning rates in male patients. The use of flexible cystoscopy after intracorporeal double-J stenting following LUL is an effective, quick, simple, and safe method that does not require position changes.

## Conclusions

Intracorporeal double-J stenting during laparoscopic ureterolithotomy is technically difficult, and malpositioning of stents often occurs after surgery. Immediate correction of double-J stent malpositioning based on outcomes of flexible cystoscopy is a simple and effective method that can be used after laparoscopic ureterolithotomy.

## Additional file

Additional file 1: Correction of Double-J stent malposition using two monitoring system. When the double-J stent was not visualized in the bladder on field of vision of flexible cystoscopy, the surgeon pushed the stent inferiorly using a laparoscopic instrument and monitor A until the stent came out through the ureteral orifice on monitor B. (AVI 1961 kb)

## Abbreviations

AUA: American Urological Association
EAU: European Association of Urology
EBL: Estimated blood loss
KUB: Kidney-ureter-bladder
LUL: Laparoscopic ureterolithotomy
SWL: Shockwave lithotripsy
URS: Ureteroscopic lithotripsy
